# Point‐of‐care semen analysis of patients with infertility via smartphone and colorimetric paper‐based diagnostic device

**DOI:** 10.1002/btm2.10176

**Published:** 2020-08-18

**Authors:** Yu‐Ting Tsao, Chung‐Yao Yang, Yun‐Chiao Wen, Ting‐Chang Chang, Koji Matsuura, Yu Chen, Chao‐Min Cheng

**Affiliations:** ^1^ Institute of Biomedical Engineering National Tsing Hua University Hsinchu Taiwan; ^2^ Department of Education Chang Gung Memorial Hospital Linkou Branch Taoyuan Taiwan; ^3^ Department of Research and Development Hygeia Touch Inc. Taipei Taiwan; ^4^ School of Traditional Chinese Medicine Chang Gung University College of Medicine Taoyuan Taiwan; ^5^ Department of Obstetrics and Gynecology Chang Gung Memorial Hospital Linkou Branch Taoyuan Taiwan; ^6^ Department of Biomedical Engineering, Faculty of Engineering Okayama University of Science Okayama Japan; ^7^ Department of Urology Chang Gung Memorial Hospital and Chang Gung University Taoyuan Taiwan

**Keywords:** infertility, MTT, paper‐based diagnostic device, point‐of‐care, semen analysis

## Abstract

Male infertility affects millions of males worldwide and is rising in prevalence due to social and environmental conditions. However, men often feel too embarrassed to receive a semen analysis in the hospital due to social stigmas. To overcome this problem, we developed a 3‐(4,5‐Dimethyl‐2‐thiazolyl)‐2,5‐diphenyl‐2H‐tetrazolium bromide test strip to distinguish semen samples with low total motile sperm concentration from those with normal motile sperm concentration. This is a point‐of‐care colorimetric semen analytical method with a one‐step, inexpensive, equipment‐free evaluation process, and adequate accuracy validated in a 42‐sample clinical trial. In this study, results were evaluated visually and with a smartphone application. Using visual observation methods, the area under the curve (AUC) was 0.71 (95% of confidence interval = 0.55–0.86; *p* = 0.021), sensitivity was 41%, specificity was 95%, positive predictive value was 90%, negative predictive value (NPV) was 59.4%, and accuracy was 67%. Using a smartphone recording and analytical system, AUC was 0.766 (95% of confidence interval = 0.612–0.92; *p* = 0.003), sensitivity was 96%, specificity was 65%, PPV was 75%, NPV was 92.9%, and accuracy was 80.9%. This work demonstrated a screening tool that could elevate semen analysis to the level of routine healthcare and provide for private, in‐home self‐assessment.

## 1 INTRODUCTION

Infertility is defined as a disease characterized by the failure to establish a clinical pregnancy after 12 months of regular sexual intercourse.^1^ The concept of describing infertility as a disease is part of a conscious effort to arouse public attention regarding the issue. The increasing prevalence of infertility has been associated with trends in delayed marriage, obesity, and stress.^2^, ^3^ Currently, nearly 200 million people worldwide suffer from infertility.^4^ Male infertility was found to be responsible for 20%–30% of all infertility cases and involved in 50% of cases overall.^5^ Furthermore, some researchers have discovered a significant decreasing trend in sperm quality over recent decades.^6^, ^7^, ^8^ Unfortunately, men often feel too embarrassed to seek help such as a semen analysis when encountering infertility issues, which sometimes leaves women to bear the burdens and pressures of infertility and infertility testing.^9^ Even though the infertility examination for men (semen analysis) is much easier and less invasive than that for women (ovulatory function test, hysterosalpingogram, and saline infusion sonohysterography, etc.), less men seek medical assistance with infertility than women.^10^ This imbalance may be attributable to cultural stigmas and men's' fears over loss of social status.^11^, ^12^ For all of these reasons, it is important to develop a point‐of‐care (POC) diagnostic device that allows men to evaluate sperm health in the privacy of their own homes.

The current diagnostic methods for examining male infertility are primarily rely on microscope‐based examination and computer‐assisted semen analysis (CASA) systems described in the World Health Organization (WHO) Laboratory manual for the examination and processing of human semen.^13^ Standard semen analysis comprises examinations of semen volume, pH, sperm concentration, count, motility, morphology, leukocyte count, and an immunobead test. Although standard methods could provide a holistic approach for determining sperm quality, such approaches require bulky and costly microscopes, equipment, and well‐trained technicians. These requirements are expensive, often costing hundreds of USD. They also limit accessibility to semen analysis in resource‐limit areas and push the process into the category of nonroutine health examinations. To meet the need for rapid, convenient, and easy‐to‐handle semen analysis tools, technologies for home‐based semen analysis have recently emerged. Most of the current home‐based semen analytical devices have relied on an image magnifier and an image interpretation application (APP) that can only return a few analytical parameters, for example, concentration, TMS, and motility. Furthermore, the plane image presentation of both the standard microscopic‐based diagnostic methods and the smartphone‐based evaluations are not useful for accurately tracking the three‐dimensional spatial motion of spermatozoa because they lack *z*‐axis observation capacity.^14^ The relatively high price of smartphone‐based analysis (usually >50 USD, but varied depending on economic environment) also presents an obstacle for continuous monitoring. Chemical‐based semen analysis tools, for example, the FertilityScore kit and Sperm check, offer a relatively inexpensive and useful alternative testing option. The FertilityScore kit is a colorimetric kit based on the reduction of blue resazurin dye by motile spermatozoa, and the Sperm check tool is a lateral flow assay based on an antigen–antibody interaction.^15^, ^16^ It is worth noting that the physical‐based methods usually detect the parameters of sperm concentration and motility separately while the chemical‐based methods usually detect motile sperm concentration.^17^ Here, we introduce a novel strategy that differs from those mentioned above. This approach, extremely inexpensive and easy‐to‐use, is a paper‐based diagnostic device capable of detecting total motile sperm concentration (TMSC) based on the mitochondrial activity of motile spermatozoa. It can be used to distinguish low TMSC semen samples from those with normal TMSC levels.

Mitochondrial activity and functionality are the keys to sperm functionality and play a critical role in many sperm functions including basic motility, acrosome reaction, and final fertilization.^18^ Studies have shown that defects in sperm mitochondria functionality may result in reduced sperm quality and male infertility.^19^, ^20^ Moreover, the activity of succinate dehydrogenase (SDH), one of the mitochondrial respiratory chain enzymes, was found to be highly correlated with sperm quality, including sperm concentration, motility, and vitality.^21^, ^22^, ^23^ Therefore, testing mitochondrial functionality, especially the activity of SDH, may be a potential approach for determining sperm quality. In a previous study, we developed a paper‐based diagnostic device for evaluating the mitochondrial activity of spermatozoa based on 3‐(4,5‐Dimethylthiazol‐2‐yl)‐2,5‐diphenyltetrazolium bromide (MTT), and used it to successfully examine the mitochondrial activity of porcine spermatozoa.^24^, ^25^ MTT is a yellow‐colored tetrazolium salt that can be reduced into purple‐colored formazan by SDH within the mitochondria of metabolically active cells. The amount of formazan produced is directly proportional to the metabolic activity of viable cells, indicating that MTT can be used as a powerful tool for providing information regarding basic sperm quality. Recent studies have shown that TMSC, which is calculated by multiplying the sperm volume by the percentage of sperm motility (% of grade a/b), may be a better parameter for determining male infertility than the parameters introduced by the WHO (2010).^26^, ^27^ Our MTT test strips determine TMSC based on mitochondrial functionality within spermatozoa, and may provide a more relevant determinant of sperm health than volume, concentration, or motility.

The goal of this study was to develop a truly POC diagnostic semen analysis device to distinguish semen samples with low TMSC from those with normal TMSC levels in order to assist men suffering from infertility issues. Here, we present a rapid, inexpensive, user‐friendly, colorimetrically based POC sperm health testing device that can be used with low sample volumes. This device was used to test fresh, unprocessed clinical semen samples, and results were compared to those from traditional, clinical, microscope‐based analysis. Based on our findings, we further demonstrate the potential for the development of an all‐in‐one semen analysis device that can simultaneously detect multiple parameters quickly and easily.

## 2 RESULTS

### 2.1 Basic characteristics

Forty‐three men (median age, 35.31 years) provided informed consent and were recruited for this study between 2018 and 2019. One patient was excluded because of macroscopic significant hematospermia. Therefore, a total of 42 semen samples from 42 different patients were obtained for this study. The low TMSC group (TMSC <20 × 10^6^ spermatozoa) included 22 patients, while the normal TMSC group (TMSC >20 × 10^6^ spermatozoa) included 20 patients and was used as the control group. The diagnosis of low TMSC (22 patients) for each study subject was confirmed by manual microscopic‐based semen analysis as recorded in each patient's medical record. The basic characteristics of these 42 samples are provided in Table 1.

**TABLE 1:** Patient characteristics

Patient group	Patient with normal TMSC	Patient with low TMSC	Total
No. of patients	20	22	42
Age (y)	31.6 ± 8.67	38.68 ± 7.90	35.31 ± 8.92
BMI (kg/m^2^)	23.38 ± 2.82	27.17 ± 3.78	25.37 ± 3.83
Systemic underlying diseases	2	4	6
Smoking history	2	7	9
Semen amount (ml)	3.73 ± 1.79	2.86 ± 1.59	3.27 ± 1.73
Semen color (Grayish‐white/yellow)	19/1	22/0	41/1
Semen viscosity (<2/>2, cm)	14/6	21/1	35/7
Sperm concentration (10^6^/ml)	78.45 ± 57.96	4.73 ± 10.36	39.83 ± 54.78
Motility (%)	48.7 ± 17.78	13 ± 21.61	30 ± 26.67
TMSC (x10^6^/ml)	132.87 ± 165.06	2.65 ± 5.04	17.69 ± 24.43
Normal morphology sperm concentration (10^6^/ml)	19.13 ± 11.39	4.45 ± 7.75	11.44 ± 12.08
WBC count (HPF)	2.78 ± 2.33	4.52 ± 8.4	3.57 ± 6.29
RBC count (HPF)	0.28 ± 0.30	0.45 ± 0.75	0.37 ± 0.58

*Note*: Value are presented as mean ± SD, or number of cases. Systemic underlying diseases include hypertension, diabetes mellitus, dyslipidemia, heart disease, kidney disease, etc.

Abbreviations: BMI, body mass index; RBC, red blood cell; TMSC, total motile sperm concentration; WBC, white blood cell.

### 2.2 MTT test strip semen analysis

**FIGURE 1:**
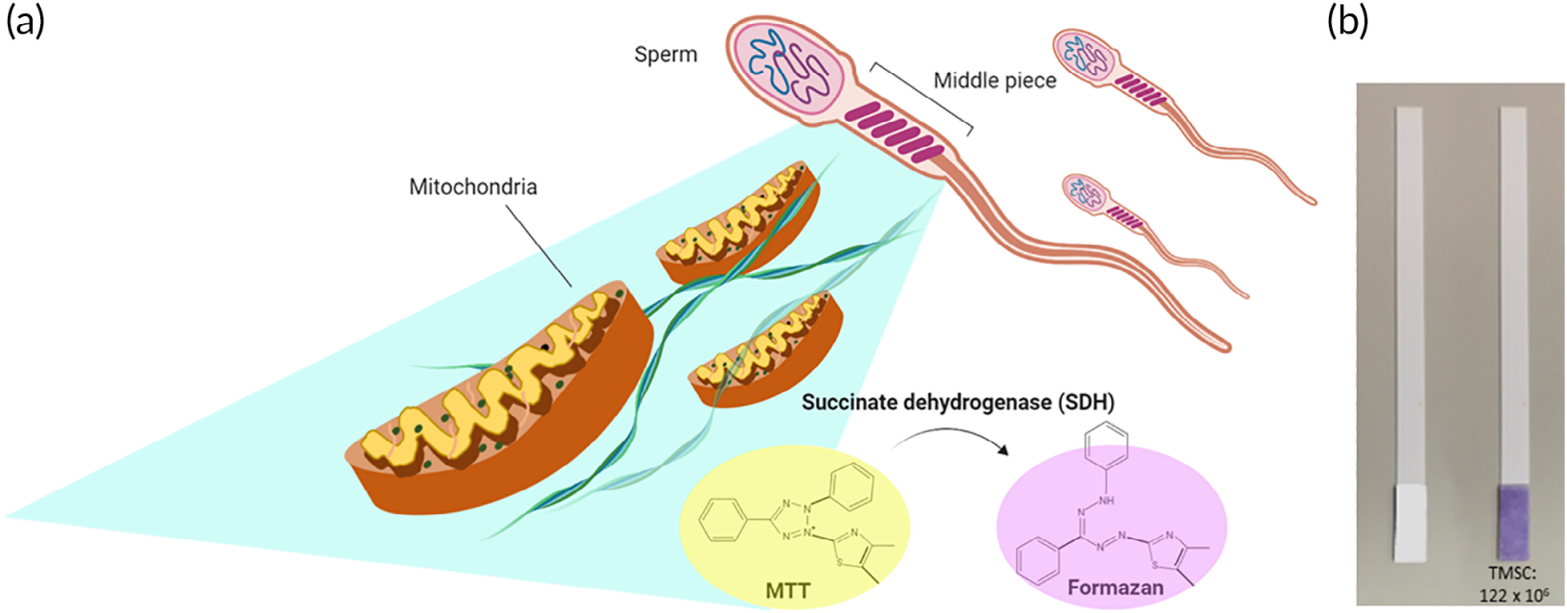
The mechanism and colorimetric results of an MTT test strip. (a) The mechanism of the sperm and MTT interaction. There is an abundance of mitochondria in the middle portion of the sperm cell, where succinate dehydrogenase (SDH) located on the mitochondria transforms yellow‐colored MTT into purple‐colored formazan. (b) The color of the MTT test strip before and after testing the semen sample. The colorimetric result is homogenously distributed over the testing zone of the MTT test strip and the result can be easily interpreted by visual observation or with a smartphone‐based recording and analytical system. MTT, 3‐(4,5‐Dimethyl‐2‐thiazolyl)‐2,5‐diphenyl‐2H‐tetrazolium bromide

**FIGURE 2:**
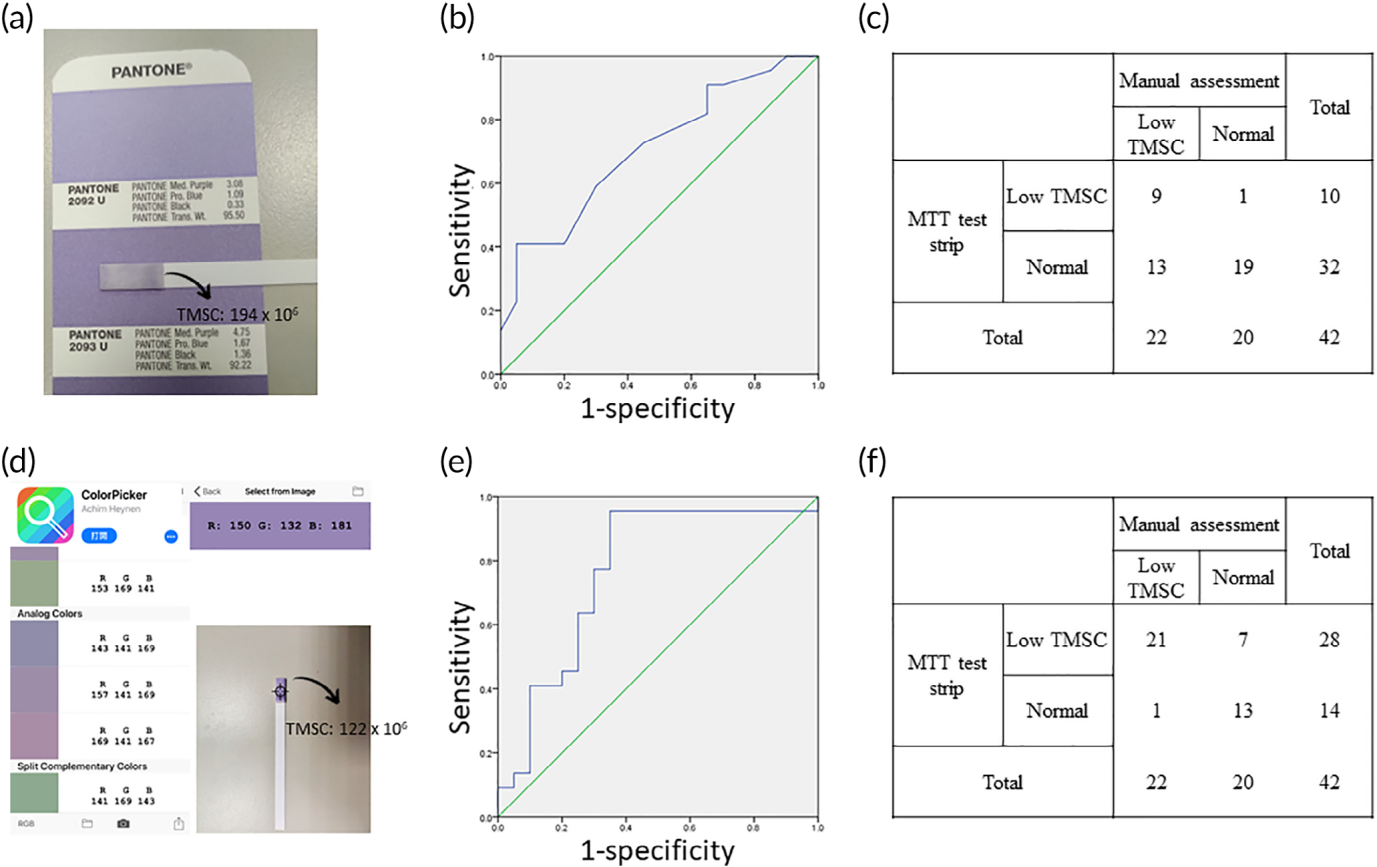
Two‐image readout system and MTT test strip results differentiate normal and low total motile sperm concentration (TMSC) semen samples. (a) The red/green/blue (RGB) colorimetric results recorded using pantone color chips (b) The receiver operating characteristic (ROC) curve based on visual observation of the MTT test strip to differentiate normal and low total motile sperm concentration (TMSC, < 20 × 10^6^ spermatozoa) in semen samples. (AUC = 0.71; 95% of confidence interval = 0.55–0.86; *p* = 0.021) (c) The confusion matrix of the results based on visual observation. (sensitivity = 41%; specificity = 95%; positive predictive value (PPV) = 90%; negative predictive value (NPV) = 59.4%; accuracy = 67%) (d) The RGB scale of the colorimetric results recorded via smartphone. The arrow in the middle part of the ColorPicker application can be used to analyze the RGB scale of the loaded picture. (e) ROC curve of an MTT test strip analyzed with the smartphone‐based recording and analytical system to differentiate normal and low TMSC semen samples. (AUC = 0.766; 95% of confidence interval = 0.612–0.92; *p* = 0.003) (f) The confusion matrix of the results recorded using the smartphone‐based recording and analytical system (sensitivity = 96%; specificity = 65%; PPV = 75%; NPV = 92.9%; accuracy = 80.9%). In figure (c), (f), the RGB threshold that separates patients predicted as normal TMSC status and low TMSC status was determined by the point on the ROC curve that maximized sensitivity and specificity. MTT, 3‐(4,5‐Dimethyl‐2‐thiazolyl)‐2,5‐diphenyl‐2H‐tetrazolium bromide

The mechanism and colorimetric change features of the MTT test strip are illustrated in Figure 1. The large number of mitochondria located in the middle portion of a sperm cell can transform yellow MTT into purple formazan via electron‐transport activity. In Figure 1(b), the colorimetric change of the test strip is significant enough for measurement using visual (naked eye) interpretation or color analyzing devices. For clinical validation of the visual, naked eye image readout system (recorded as the pantone color, illustrated in Figure 2(a) and Table SI), the colorimetric results recorded by MTT test strip were significantly correlated with TMSC (rho = 0.36, *p* = 0.019; calculated by Spearman correlation coefficient). The predictive value of all parameters mentioned in Table 1, including age, BMI, systemic underlying diseases, smoking history, and manual semen analytical parameters, were calculated by univariate logistic regression, and parameters with p‐values <0.05 were considered as confounding factors (Table 2). Subsequent multivariate logistic regression analysis indicated that the odds ratio of the MTT test strip was 0.953 (95% confidence interval = 0.902–1.002; *p* = 0.094) after adjusting for confounding factors (age, BMI, and normal morphological sperm concentration). We obtained an area under the curve (AUC) of the receiver operating characteristic (ROC) curve of 0.71 (95% confidence interval = 0.55–0.86; *p* = 0.021) when the MTT test strip was compared with conventional semen analysis (Figure 2(b)). The red green blue (RGB) threshold that separated patients diagnosed with normal TMSC status and low TMSC status was determined by the point on the ROC curve that maximized sensitivity and specificity. The sensitivity, specificity, positive predictive value (PPV), negative predictive value (NPV), and accuracy were obtained at the cut‐off value of 20x10^6^ TMSC, which gave the results of 41%, 95%, 90%, 59.4%, and 67%, respectively. These data were presented in the confusion matrix (Figure 2(c)). A smartphone‐based system, a general smartphone and a free APP (ColorPicker) were used to assist the color interpretation (Figure 2(d)). We used the ColorPicker APP to transform the colorimetric changes of the test strip into RGB scale. After adjusting for the “blank” color, colorimetric results were converted to grayscale for analysis. The analytical data for all the patients are illustrated in Table SII. The results were significantly correlated to TMSC (rho = 0.452, *p* = 0.003; calculated by Spearman correlation coefficient). Using this approach, the odds ratio of the MTT test strip for predicting male fertility status was 0.947 (95% confidence interval = 0.904–0.991; *p* = 0.02, calculated by multivariate logistic regression analysis) after adjusting for confounding factors including age, BMI, and normal morphological sperm concentration. The AUC of the ROC was 0.766 (95% confidence interval = 0.612–0.92; p = 0.003) as demonstrated in Figure 2(e). The sensitivity, specificity, PPV, NPV, and accuracy were calculated as 96%, 65%, 75%, 92.9%, and 80.9%, respectively. These data were also presented in the confusion matrix (Figure 2(f)).

**TABLE 2:** Predictive value of the parameters mentioned in Table 1

Age (y)	^a^ *p* = 0.015, Exp (B) = 1.116
BMI (kg/m^2^)	^a^ *p* = 0.005, Exp (B) = 1.397
Systemic underlying disease	*p* = 0.900
Smoking history	*p* = 0.101
Semen color (Grayish‐white/yellow)	*p* = 1
Semen viscosity (<2 / >2, cm)	*p* = 0.053
Normal morphology sperm concentration(10^6^/ml)	^a^ *p* = 0.001, Exp (B) = 0.825
WBC count (HPF)	*p* = 0.477
RBC count (HPF)	*p* = 0.356

^a^ Parameters with significant predictive value.

## 3 DISCUSSION

This study introduced a POC semen analytical test strip that can distinguish semen samples with low TMSC from those with normal TMSC levels by detecting the amount of mitochondrial activity in sperm. The results were generated and observable via a colorimetric change, that is, yellow MTT to purple formazan. The experiment extended previous studies suggesting that a paper‐based MTT assay could be used to monitor sperm quality.^24^, ^25^ We further compared our results with those from standard microscopic‐based semen analyses and found that the results from using our MTT test strip were significantly correlated with TMSC. The MTT test strip can distinguish low male TMSC status (TMSC <20 × 10^6^ spermatozoa) from normal TMSC status with an accuracy of 67% using just a visual, naked eye image readout system and with an accuracy of 80.9% using a smartphone‐based system. The smartphone‐based recording and analytical system showed superior predictive ability for determining male infertility compared to the naked eye image readout system, which may be attributed to the more accurate RGB analytical capacity of the smartphone. The ColorPicker APP can precisely transform the colorimetric image to its corresponding RGB values and perfectly distinguish shades of color. This is a significant advantage over the naked eye system, which relies on human‐made comparisons to pantone color chips. Altogether, the standardized protocol of the smartphone‐based system avoids subjective factors and significantly improves diagnostic accuracy. In addition, it is worth noting that the sensitivity of the MTT test strip when using the smartphone‐based system was 96%, indicating that the MTT test strip can be adopted as a suitable screening tool for POC semen analysis in a variety of circumstances.

**FIGURE 3:**
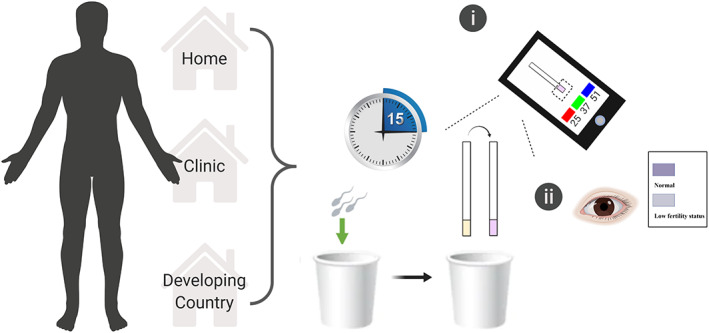
Potential clinical application of the MTT test strip. The equipment‐free MTT test strip allows men to evaluate their semen quality anywhere, whether in the home, the clinic, or in resource‐limited developing countries. After collecting a semen sample, men can easily determine results using a smartphone or color indicator chart (within 15 min). MTT, 3‐(4,5‐Dimethyl‐2‐thiazolyl)‐2,5‐diphenyl‐2H‐tetrazolium bromide

To select an appropriate diagnostic tool, one can follow the ASSURED criteria provided by WHO.^28^ In keeping with this set of criteria, the MTT test strip we developed displayed characteristics of being affordable (approximately 0.03 USD per test), user‐friendly (1 step), rapid (less than 30 min), equipment‐free, and deliverable to end‐users. The MTT test strip allows men to evaluate their semen quality at home, in clinics, or in developing countries where infertility problems are severe, but sophisticated equipment and well‐trained technicians are difficult to access.^29^ Moreover, because MTT test strips are easy to use and cost‐effective, they could facilitate and promote the inclusion of semen analysis in routine health examinations. Men who suffer from infertility or have undergone a vasectomy can easily use the MTT test strip as a screening or follow‐up tool. This would provide a clear benefit to men, but would also reduce stress on women who are typically burdened with the responsibility for fertility testing. A schematic diagram demonstrating the applied use of an MTT test strip is provided in Figure 3.

**FIGURE 4:**
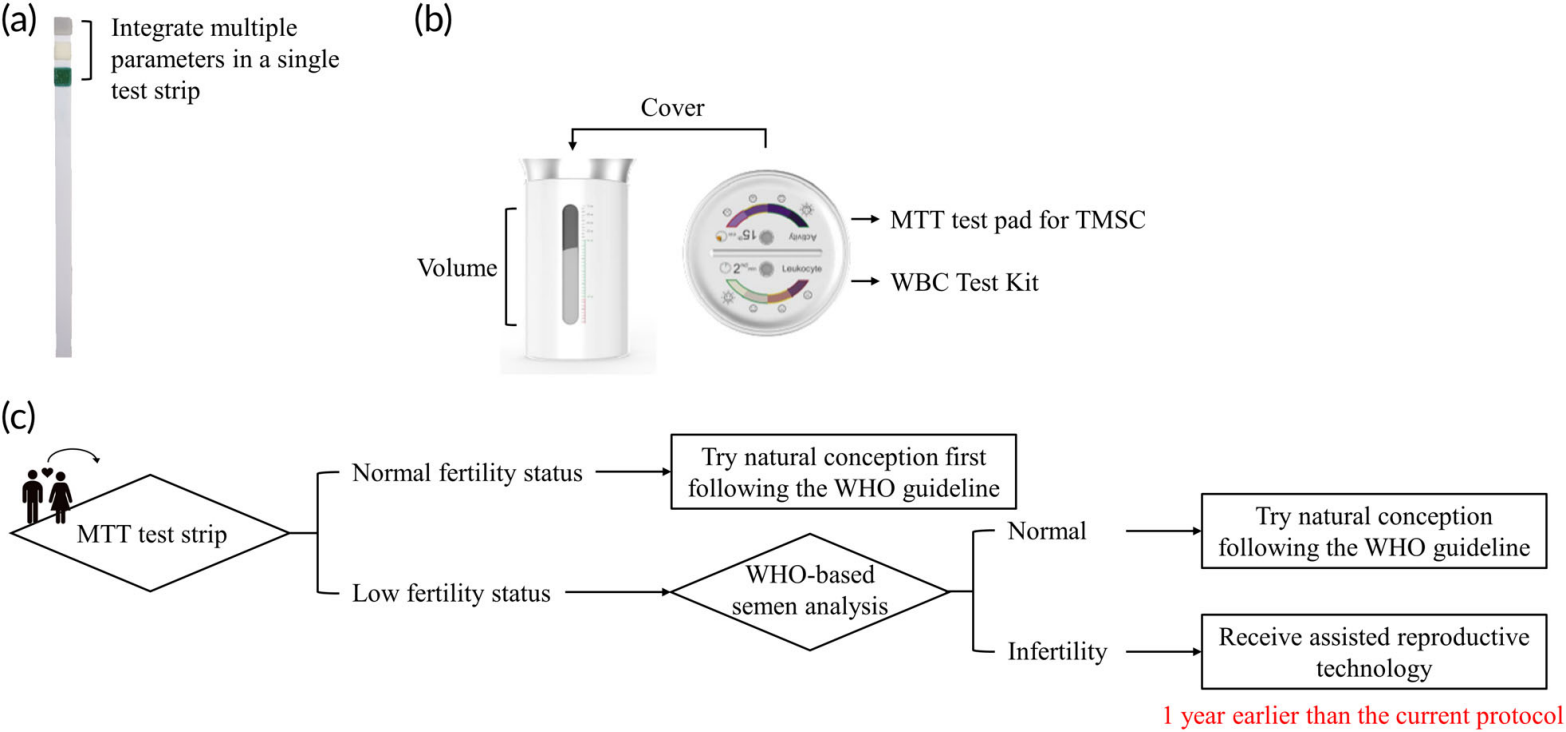
Potential cotton‐based holistic semen analytical devices and MTT test strip application flow chart (a) The test strip design allows integration of multiple parameters in a single test. The co‐tested parameters can include pH, white blood cell count, and other parameters. (b) The transformable property of the cotton‐based MTT test strip allows for redesign. Used in combination, the measuring cylinder and the MTT test pad attached to the cover could provide more information about semen quality. (c) This flow chart suggests that men evaluate their semen quality before trying to conceive. If the result shows normal total motile sperm concentration (TMSC) status, men can try natural conception first, following the WHO guidelines, but if the result shows low TMSC status, we suggest that men go to the hospital for a detailed examination. Following this flow chart, infertile man can receive appropriate intervention earlier than they would under the current WHO guidelines, which suggests that couples attempt normal conception for 1 year before having any examination. MTT, 3‐(4,5‐Dimethyl‐2‐thiazolyl)‐2,5‐diphenyl‐2H‐tetrazolium bromide

An estimated 15% of all couples suffer from infertility,^30^, ^31^ and 10% of all couples rely on vasectomy for contraception.^32^, ^33^ As a result, at least 25% of all couples worldwide require semen analysis. The high demand for semen analysis has given rise to a variety of POC semen analytical devices. The Fertell test, developed in 2006, was the world's first commercially available home‐based semen analytical device.^34^ In this device, a layer of hyaluronic acid is used to select progressively motile spermatozoa through a swim‐up process, and the concentration of progressively motile sperm is measured by lateral flow immunoassay. Overall, POC semen analytical tool methodology can be divided into chemical‐based semen analytical devices, physical‐based semen analytical devices, and combined‐system devices. The chemical‐based semen analytical methods include colorimetric reaction and lateral flow immunochromatography, which can measure sperm concentration or TMSC.^15^, ^16^ The chemical‐based semen analytical methods are characterized by low cost and their lack of equipment needs. The physical‐based semen analytical methods include centrifugation^35^, ^36^ and magnification. In the magnification method, sperm images can be magnified by a miniaturized microscope^37^ or a portable optical attachment, and images can be analyzed using a computer or smartphone.^38^, ^39^, ^40^ The physical‐based semen analytical methods can be used to determine sperm concentration and motility in a quantitative manner and they can do so with high accuracy. Some semen analytical devices combine chemical and physical methodologies into a single device that provides information provided by both approaches.^17^, ^41^, ^42^, ^43^ Comparative details of current POC semen analytical methodologies are provided in Table 3. The MTT test strip we developed is a cotton‐based diagnostic tool that appears to be the most inexpensive and easy‐to‐use semen analytical device, with a cost of less than 0.03 USD per test (about 1,500 times cheaper than the cost of currently available semen analytical devices). This approach requires only a single step, compared to the 2 to 4 steps required by other currently available semen analytical devices. Although the accuracy of the MTT test strip is relatively low (67%–81%) compared to currently available POC semen analytical devices, the high sensitivity (up to 96%) of the MTT test strip makes it a suitable semen quality screening tool capable of diagnosing possible infertility issues. In addition, paper‐based or cotton‐based analytical devices appear to be the most promising platform for developing physiological fluid‐based diagnostic devices because of their low‐cost, portability, trace substance requirements, and adaptability.^44^, ^45^ The adaptability of these paper‐based or cotton‐based diagnostic devices allows them to overcome some of the limitations of currently available, at‐home semen analytical tools, such as their bulky size and limited parameter testing. The design of our MTT test strip enables several determinations simultaneously. The potential for cotton‐based semen analytical devices is summarized in Figure 4(a),(b). These devices facilitate measurements of volume, TMSC, white blood cell count, pH level, and other parameters in a single device and with only a single step.

**TABLE 3:** A comparison of current point‐of‐care semen analytical methodology

		Chemical‐based semen analytical method		Physical‐based semen analytical method			Combination of physical‐ and chemical‐based analytical method		
Mechanism	Colorimetric reaction		Lateral flow immuno‐chromatography	Centrifugation	Magnified image by miniaturized microscope	Magnified and record image by optical attachment and smartphone	Combined (motility filter and centrifugal force)	Motility filter and colorimetric reaction	Motility filter and lateral flow immunoassay
Parameters tested	TMSC	Motile SC	SC	SC, volume	SC, motility	SC, motility	SC, motility	SC, motility	Motile SC
Accuracy	67%–81%	86%	95%–96%	93%	‐	97%	‐	72.4%–100%	95%
Time requirement	15 min	90 min	7–12 min	6 min	10 s	4.48 s–13 min	35 min	10–30 min	30 min
Equipment requirement	No	No	No	Engine	Microscope, computer	Smartphone, optical magnifier	Centrifuge, microscope	No/letter‐sized scanner	No
Cost/test	$0.03	$12.5	$39.99–$48	$74.99	$74.49	$4.45–$69.95	‐	$0.05–$55	‐
Step	1	3	3	3	2–3	3–4	3	2–3	2
Representative products	MTT test strip	Fertility score kit^17^, ^57^	Sperm check^16^ sperm OK	Trak male fertility testing System^35^	Micra sperm test lens‐free on‐chip microscopy by TW Su^38^	Yo Sperm^40^ smartphone‐based diagnostic assay by MK Kanakasabapathy^39^	Microfludic device by CY Chen and TC Chiang^36^	Swimcount^42^ paper‐based semen analysis device by Nosrati R and Gong MM^43^	Fertell sperm test^34^

Abbreviations: MTT, 3‐(4,5‐Dimethyl‐2‐thiazolyl)‐2,5‐diphenyl‐2H‐tetrazolium bromide; SC, sperm concentration; TMSC, total motile sperm count.

As highlighted above, the MTT test strip provides convenient and rapid qualitative TMSC values based on a measurement of mitochondrial activity. Because the age at which people are getting married has been increasing in many countries,^46^, ^47^ early detection of possible infertility factors is an important issue for early fertility management and better pregnancy outcomes. Early evaluation of semen quality by current WHO manual or CASA methods is labor‐intensive, expensive, and time‐consuming. Men who want to conceive a child would benefit by the availability of MTT test strip technology for early, home‐based screening. If results indicate normal TMSC status, men can try natural conception following the WHO guidelines; but if results indicate low TMSC status, we suggest that men go to the hospital for a more detailed examination, and further determination regarding whether they should receive assisted reproductive technology (Figure 4(c)). Following this process may help men with infertility to receive appropriate intervention earlier than the current WHO guidelines, which suggests that couples try normal conception for 1 year before having any examination. Due to the high sensitivity and low cost of the MTT test strip, we believe it could facilitate more rapid and effective management of possible infertility issues. However, it should be noted that we do not suggest using the MTT test strip to replace standard semen analysis. If MTT test strips indicate normal TMSC, but couples fail to conceive over a year's time, they should seek help following the WHO guidelines.

Despite its clear value, this process has limitations. First, the sample size was relatively small, and the sample source was restricted to Asian men. Further, although we have validated our experiment using spiking methods under very controlled conditions and animal testing in our previous study, the optimized concentration of MTT required for manufacturing the test strip is different for human samples compared to the porcine samples we used in our previous study.^24^, ^25^ A greater number of samples, and a selection of samples from additional races could provide advantageous results and further validation. Another issue is the lack of a clinical test value directly comparable to the mitochondrial activity tested using the MTT test strip. Clinical tests and medical reports based on the WHO manual methods could only provide the values of volume, turbidity, color, viscosity, sperm count, motility, vitality, morphology, white blood cell count, and red blood cell count. Furthermore, many studies have shown that the current WHO criteria for semen analysis are not enough for adequate determination of fertility level.^48^, ^49^, ^50^, ^51^ The poor prognostic power of the current diagnostic criteria may result from their lack of functional consideration of spermatozoa characteristics including DNA integrity and mitochondrial activity, which are key to successful fertilization.^52^, ^53^, ^54^ In our study, we could only choose the most correlated parameter, that is, TMSC, which examined multiple parameters to increase the potential for determining fertilization.^26^, ^27^ However, a specialized study to evaluate the correlation between MTT test strip results and spermatozoan mitochondrial normality may be required. Furthermore, to truly validate the predictive ability of the MTT test strip for determining male infertility, a meticulous study design with a longer follow‐up period lasting for at least 1 year would facilitate the tracking of real fertility outcomes and may yield more significant results. Finally, the results of the MTT test strip may be interfered with by the presence of other karyocytes, for example, white blood cells with mitochondria, that could also transform yellow MTT into purple formazan. This limitation may be overcome with the use of a filter or by simultaneous determination of WBC count as shown in Figure 4(a),(b).

## 4 MATERIALS AND METHODS

### 4.1 Study design and patients

This is a cross‐sectional study to assess the utility of our MTT test strip for examining clinical samples and comparing them to clinically derived results, that is, microscopy‐based, gold standard semen analysis. Patients receiving semen analysis due to infertility concerns were recruited from the Division of Urology at Chang Gung Memorial Hospital, Linkou. Patients with extremely low semen volume, that is, levels that were insufficient to complete the test and those with hematospermia, were excluded. This study was approved by the local institutional review board of Chang Gung Memorial Hospital in 2018 (IRB number: 201800088B0) and followed the relevant guidelines. Informed consent was obtained from all patients after providing them with a detailed explanation of the study. All semen samples were collected from March 2018 to July 2019 (n = 43).

### 4.2 Semen preparation

The semen samples were provided via patient masturbation following 3 to 5 days of sexual abstinence. The semen samples were ejaculated into an aseptic plastic cup and shelved for 30 min at room temperature for semen liquefaction.^13^ All semen samples were obtained using sterilized cups and analyzed using our MTT test strips immediately following completion of clinical semen analysis. The time span between ejaculation and both the manual semen analysis and the MTT strip test was less than 1 h.

### 4.3 MTT test strip fabrication

The test strip consisted of a ribbon made of paper and a test pad made of cotton adhered to the tip of each strip. To encourage user‐friendliness in our POC diagnostic device, the test pad was designed with a width of approximately 5 mm and a length of 10 mm. MTT purchased from Sigma‐Aldrich (No.: M2128, USA) was used as the coating reagent. To manufacture our MTT test strip, test pads were impregnated with a solution of 0.75 mM MTT and 5% sucrose, which was used as a stabilizer.^55^, ^56^ The MTT test strip was subsequently dried at room temperature overnight. While drying, it was kept in the shade, at room temperature, and away from light. If kept away from light, humidity, and heat, the test strip can be stored and used for more than a year.

### 4.4 Experimental section: Semen analysis

To execute semen analysis, MTT test strips were immersed directly into liquefied semen samples for approximately 2 s. The test strip was subsequently removed from the semen sample and placed on a horizontal surface for a 15 min reaction period away from the light. During this time, the reactive SDH found within sperm mitochondria transformed the yellow‐colored MTT into purple‐colored formazan. The resulting colorimetric change was easily captured and analyzed. To achieve the goal of developing a true POC semen analytical device, we carried out two simple strategies to interpret the colorimetric results without sophisticated equipment: (a) a visual, naked eye comparison; and, (b) a smartphone‐based recording and analysis. When employing the visual, naked eye assay, we compared the postreaction test strip color with pantone color chips to find the most similar match. We recorded the RGB scale of the selected color chips. The difference between the pantone color chips and the blank value (white, RGB 255; 255; 255) was calculated for further analysis. When using the smartphone‐based system, we recorded the intensity of the purple signal using the camera of a simple smartphone (Asus Zenfone 6, Taiwan), and then processed the colorimetric results using an RGB detection APP, ColorPicker. This APP can be installed via the App Store or via Google Play. We used the free version. The central area of the test strip was marked as the tested point and the white paper strip area near the tested pad area was marked as the blank value. The difference in RGB values between the tested point and the blank value was recorded for further analysis. All procedures were carried out at room temperature, and the experimental time from sample preparation to image analysis was less than 1 h. Only an MTT test strip and a smartphone were required to complete this test.

The MTT test strip results were compared to clinically determined TMSC results from patient medical records. Clinical tests were performed and recorded using the manual, microscope‐based method described in the WHO criteria (2010), which is considered the gold standard.^13^ In this experiment, TMSC values of more than 20 × 10^6^ spermatozoa were defined as being in the range of normal TMSC status, while TMSC values below 20 × 10^6^ spermatozoa were considered to be indicative of low TMSC status in accordance with the reference mentioned above.^26^, ^27^

### 4.5 Statistical analysis

Statistical analyses were performed using software (IBM SPSS Statistics for Windows, Version 22.0. Armonk, NY: IBM Corp.). A p‐value of less than 0.05 was considered statistically significant. The colorimetric results of the MTT strip test were converted into grayscale where gray = (red + green + blue). For continuous data, the normality of the distribution was tested using the Shapiro–Wilk test. Pearson's correlation coefficient was used to calculate the normally distributed data, and Spearman correlation analysis was used for the nonnormal distributed data. For results with significant correlation, multivariate logistic regression analysis was used to assess the predictive probability of male infertility using our MTT‐test strip, and results were demonstrated using a ROC curve with AUC and a 95% confidence interval. Based on the ROC curve, the Youden's J statistic was used to calculate the maximum potential effectiveness of the MTT test strip. The results were presented as a confusion matrix, wherein we calculated sensitivity, specificity, PPV, NPV, and accuracy.

## 5 CONCLUSIONS

This study demonstrates a rapid, inexpensive, sensitive, equipment‐free, and easy‐to‐use POC semen analytical tool for assessing male fertility. Men can easily test their semen quality with a one‐step evaluation process at home using the MTT test strip, which costs only $0.03 per test. Our results were clinically validated by comparing them to TMSC values from the medical records of 42 patients. As the problem of infertility becomes an increasingly pressing issue, implementation of a tool such as our MTT test strip could alleviate burdens and increase the efficiency of fertility testing and planned pregnancy, speeding the process by as much as an entire year compared to the generally accepted protocol.

## 6 CONFLICT OF INTEREST

The authors declare no potential conflict of interest

## Supporting information


**Table SI** Colorimetric result of the semen sample recorded by naked eye and pantone color chips and the corresponding total motile sperm count

**Table SII** Colorimetric result of the semen sample interpreted by the smartphone and the corresponding total motile sperm count

## References

[1] Zegers‐Hochschild F , Adamson GD , Dyer S , et al. The international glossary on infertility and fertility care, 2017. Fertil Steril. 2017;108(3):393‐406. 10.1016/j.fertnstert.2017.06.005. 28760517

[2] Bunting L , Boivin J . Knowledge about infertility risk factors, fertility myths and illusory benefits of healthy habits in young people. Hum Reprod. 2008;23(8):1858‐1864. 10.1093/humrep/den168. 18469334

[3] Skakkebaek NE , Rajpert‐De Meyts E , Buck Louis GM , et al. Male reproductive disorders and fertility trends: influences of environment and genetic susceptibility. Physiol Rev. 2015;96(1):55‐97. 10.1152/physrev.00017.2015. PMC4698396 26582516

[4] Inhorn MC , Patrizio P . Infertility around the globe: new thinking on gender, reproductive technologies and global movements in the 21st century. Hum Reprod Update. 2015;21(4):411‐426. 10.1093/humupd/dmv016. 25801630

[5] Vander Borght M , Wyns C . Fertility and infertility: definition and epidemiology. Clin Biochem. 2018;62:2‐10. 10.1016/j.clinbiochem.2018.03.012. 29555319

[6] Huang C , Li B , Xu K , et al. Decline in semen quality among 30,636 young Chinese men from 2001 to 2015. Fertil Steril. 2017;107(1):83‐88.e2. 10.1016/j.fertnstert.2016.09.035. 27793371

[7] Sengupta P , Borges E , Dutta S , Krajewska‐Kulak E . Decline in sperm count in European men during the past 50 years. Hum Exp Toxicol. 2018;37(3):247‐255. 10.1177/0960327117703690. 28413887

[8] Centola GM , Blanchard A , Demick J , Li S , Eisenberg ML . Decline in sperm count and motility in young adult men from 2003 to 2013: observations from a U.S. sperm bank. Andrology. 2016;4(2):270‐276. 10.1111/andr.12149. 26789272

[9] Culley L , Hudson N , Lohan M . Where are all the men? The marginalization of men in social scientific research on infertility. Reprod Biomed Online. 2013;27(3):225‐235. 10.1016/j.rbmo.2013.06.009. 23871364

[10] Datta J , Palmer MJ , Tanton C , et al. Prevalence of infertility and help seeking among 15 000 women and men. Hum Reprod. 2016;31(9):2108‐2118. 10.1093/humrep/dew123. 27365525 PMC4991655

[11] Dyer SJ , Abrahams N , Mokoena NE , van der Spuy ZM . 'You are a man because you have children': experiences, reproductive health knowledge and treatment‐seeking behaviour among men suffering from couple infertility in South Africa. Hum Reprod. 2004;19(4):960‐967. 10.1093/humrep/deh195. 15016772

[12] Smith JF , Walsh TJ , Shindel AW , et al. Sexual, marital, and social impact of a man's perceived infertility diagnosis. J Sex Med. 2009;6(9):2505‐2515. 10.1111/j.1743-6109.2009.01383.x. 19619144 PMC2888139

[13] World Health Organization . WHO Laboratory Manual For The Examination And Processing Of Human Semen. 5th ed. Geneva: World Health Organization; 2010.

[14] Su T‐W , Xue L , Ozcan A . High‐throughput lensfree 3D tracking of human sperms reveals rare statistics of helical trajectories. Proc Natl Acad Sci. 2012;109(40):16018‐16022. 10.1073/pnas.1212506109. 22988076 PMC3479566

[15] Mahmoud A , Vermeulen L , Schoonjans F , Comhaire F . Performance of the fertility score kit for home semen analysis. Human Reproduction. 1994;9:110‐137.8195331

[16] Coppola M , Klotz K , K‐a K , et al. SpermCheck® fertility, an immunodiagnostic home test that detects normozoospermia and severe oligozoospermia. Hum Reprod. 2010;25(4):853‐861. 20139122 10.1093/humrep/dep413 PMC2839906

[17] Kobori Y . Home testing for male factor infertility: a review of current options. Fertil Steril. 2019;111(5):864‐870. 30922654 10.1016/j.fertnstert.2019.01.032

[18] Moraes CR , Meyers S . The sperm mitochondrion: organelle of many functions. Anim Reprod Sci. 2018;194:71‐80. 10.1016/j.anireprosci.2018.03.024. 29605167

[19] Song GJ , Lewis V . Mitochondrial DNA integrity and copy number in sperm from infertile men. Fertil Steril. 2008;90(6):2238‐2244. 10.1016/j.fertnstert.2007.10.059. 18249386

[20] Spiropoulos J , Turnbull DM , Chinnery PF . Can mitochondrial DNA mutations cause sperm dysfunction? Mol Hum Reprod. 2002;8(8):719‐721. 10.1093/molehr/8.8.719. 12149402

[21] Ruiz‐Pesini E , Lapeña AC , Díez C , Álvarez E , Enríquez JA , López‐Pérez MJ . Seminal quality correlates with mitochondrial functionality. Clin Chim Acta. 2000;300(1):97‐105. 10.1016/S0009-8981(00)00305-3. 10958866

[22] Ruiz‐Pesini E , Diez C , Lapeña AC , et al. Correlation of sperm motility with mitochondrial enzymatic activities. Clin Chem. 1998;44(8):1616‐1620. 9702947

[23] Alexandra A , Bárbara L , Mónica M , João R‐S . Mitochondria functionality and sperm quality. Reproduction. 2013;146(5):R163‐R174. 10.1530/REP-13-0178. 23901129

[24] Matsuura K , Chen KH , Tsai CH , et al. Paper‐based diagnostic devices for evaluating the quality of human sperm. Microfluid Nanofluid. 2014;16(5):857‐867. 10.1007/s10404-014-1378-y.

[25] Matsuura K , Huang HW , Chen MC , Chen Y , Cheng CM . Relationship between porcine sperm motility and sperm enzymatic activity using paper‐based devices. Sci rep. 2017;7:46213 10.1038/srep46213. 28387379 PMC5384208

[26] Borges E Jr , Setti AS , Braga DPAF , Figueira RCS , Iaconelli A Jr . Total motile sperm count has a superior predictive value over the WHO 2010 cut‐off values for the outcomes of intracytoplasmic sperm injection cycles. Andrology. 2016;4(5):880‐886. 10.1111/andr.12199. 27152971

[27] Hamilton JAM , Cissen M , Brandes M , et al. Total motile sperm count: a better indicator for the severity of male factor infertility than the WHO sperm classification system. Hum Reprod. 2015;30(5):1110‐1121. 10.1093/humrep/dev058. 25788568

[28] Kosack CS , Page A‐L , Klatser PR . A guide to aid the selection of diagnostic tests. Bull World Health Organ. 2017;95(9):639‐645. 28867844 10.2471/BLT.16.187468 PMC5578377

[29] Ombelet W . Global access to infertility care in developing countries: a case of human rights, equity and social justice. Facts Views Obgyn. 2011;3(4):257‐266. PMC3987469 24753875

[30] Agarwal A , Mulgund A , Hamada A , Chyatte MR . A unique view on male infertility around the globe. Reprod Biol Endocrinol. 2015;13:37‐37. 10.1186/s12958-015-0032-1. 25928197 PMC4424520

[31] Kumar N , Singh AK . Trends of male factor infertility, an important cause of infertility: a review of literature. J Hum Reprod Sci. 2015;8(4):191‐196. 10.4103/0974-1208.170370. 26752853 PMC4691969

[32] Ostrowski KA , Holt SK , Haynes B , Davies BJ , Fuchs EF , Walsh TJ . Evaluation of vasectomy trends in the United States. Urology. 2018;118:76‐79. 10.1016/j.urology.2018.03.016. 29578040

[33] Jacobstein R . The kindest cut: global need to increase vasectomy availability. Lancet Glob Health. 2015;3(12):e733‐e734. 26545447 10.1016/S2214-109X(15)00168-0

[34] Björndahl L , Kirkman‐Brown J , Hart G , Rattle S , Barratt CLR . Development of a novel home sperm test. Hum Reprod. 2005;21(1):145‐149. 10.1093/humrep/dei330. 16267078

[35] Schaff UY , Fredriksen LL , Epperson JG , et al. Novel centrifugal technology for measuring sperm concentration in the home. Fertil Steril. 2017;107(2):358‐364.e4. 10.1016/j.fertnstert.2016.10.025. 27887718

[36] Chen C‐Y , Chiang T‐C , Lin C‐M , et al. Sperm quality assessment via separation and sedimentation in a microfluidic device. Analyst. 2013;138(17):4967‐4974. 10.1039/C3AN00900A. 23817531

[37] Brezina PR , Haberl E , Wallach E . At home testing: optimizing management for the infertility physician. Fertil Steril. 2011;95(6):1867‐1878. 10.1016/j.fertnstert.2011.01.001. 21324446

[38] Su TW , Erlinger A , Tseng D , Ozcan A . Compact and light‐weight automated semen analysis platform using lensfree on‐chip microscopy. Anal Chem. 2010;82(19):8307‐8312. 10.1021/ac101845q. 20836503 PMC2987715

[39] Kanakasabapathy MK , Sadasivam M , Singh A , et al. An automated smartphone‐based diagnostic assay for point‐of‐care semen analysis. Sci Transl Med. 2017;9(382):eaai7863 10.1126/scitranslmed.aai7863. 28330865 PMC5701517

[40] Agarwal A , Panner Selvam MK , Sharma R , et al. Home sperm testing device versus laboratory sperm quality analyzer: comparison of motile sperm concentration. Fertil Steril. 2018;110(7):1277‐1284. 10.1016/j.fertnstert.2018.08.049. 30424879

[41] Yoon YE , Kim TY , Shin TE , et al. Validation of SwimCount™, a novel home‐based device that detects progressively motile spermatozoa: correlation with World Health Organization 5th semen analysis. World J Mens Health. 2020 Apr;38(2):191‐197. 10.5534/wjmh.180095. 30799559 PMC7076315

[42] Castello D , Garcia‐Laez V , Buyru F , Bakiricioglu E , Ebbesen T , et al. Comparison of the SwimCount home diagnostic test with conventional sperm analysis. Adv Androl Gynecol. 2018;2018:1‐5. 10.29011/AAG-101.

[43] Nosrati R , Gong MM , San Gabriel MC , Pedraza CE , Zini A , Sinton D . Paper‐based quantification of male fertility potential. Clin Chem. 2016;62(3):458‐465. 10.1373/clinchem.2015.250282. 26747445

[44] Lin SC , Hsu MY , Kuan CM , et al. Cotton‐based diagnostic devices. Sci rep. 2014;4:6976‐6976. 10.1038/srep06976. 25393975 PMC5382709

[45] Syedmoradi L , Gomez FA . Paper‐based point‐of‐care testing in disease diagnostics. Bioanalysis. 2017;9(11):841‐843. 10.4155/bio-2017-0080. 28644046

[46] Payne KK . Median age at first marriage, 2017. Family Profiles. 2019 10.25035/ncfmr/fp-19-06.

[47] Yu J , Xie Y . Changes in the determinants of marriage entry in post‐reform urban China. Demography. 2015;52(6):1869‐1892. 26391287 10.1007/s13524-015-0432-z

[48] Van Der Steeg JW , Steures P , Eijkemans MJC , et al. Role of semen analysis in subfertile couples. Fertil Steril. 2011;95(3):1013‐1019. 10.1016/j.fertnstert.2010.02.024. 20338556

[49] Ford WCL . Comments on the release of the 5th edition of the WHO Laboratory manual for the examination and processing of human semen. Asian J Androl. 2010;12(1):59‐63. 10.1038/aja.2008.57. 20111082 PMC3739684

[50] Esteves SC . Clinical relevance of routine semen analysis and controversies surrounding the 2010 World Health Organization criteria for semen examination. Int Braz J Urol. 2014;40:433‐453. 25254609 10.1590/S1677-5538.IBJU.2014.04.02

[51] Eliasson R . Semen analysis with regard to sperm number, sperm morphology and functional aspects. Asian J Androl. 2010;12(1):26‐32. 10.1038/aja.2008.58. 20111078 PMC3739674

[52] Agarwal A , Cho CL , Esteves SC . Should we evaluate and treat sperm DNA fragmentation. Current Opinion Obstetrics Gynecol. 2016;28(3):164‐171. 10.1097/gco.0000000000000271. 27054510

[53] Borini A , Tarozzi N , Nadalini M . Sperm DNA fragmentation testing in male infertility work‐up: are we ready? Transl Androl Urol. 2017;6(4):S580‐S582. 10.21037/tau.2017.03.81. 29082181 PMC5643714

[54] Bisht S , Faiq M , Tolahunase M , Dada R . Oxidative stress and male infertility. Nat Rev Urol. 2017;14:470‐485. 10.1038/nrurol.2017.69. 28508879

[55] Yüksel N , Baykara T . Preparation of polymeric microspheres by the solvent evaporation method using sucrose stearate as a droplet stabilizer. J Microencapsul. 1997;14(6):725‐733. 10.3109/02652049709006822. 9394253

[56] Sahagian ME , Goff HD . Thermal, mechanical and molecular relaxation properties of stabilized sucrose solutions at sub‐zero temperatures. Food Res Int. 1995;28(1):1‐8. 10.1016/0963-9969(95)93324-N.

[57] FertilityScore kit. FertiPro. Accessed July 14, 2020, https://fertipro.com/2019/11/13/fertilityscore-kit/

