# Supplementary material for: Point‐of‐care semen analysis of patients with infertility via smartphone and colorimetric paper‐based diagnostic device
Source: Bioeng Transl Med. 2020 Aug 18;6(1):e10176. doi: 10.1002/btm2.10176 (PMC7823130; doi:10.1002/btm2.10176)
Supplement: Supplementary file 1 — Table SI Colorimetric result of the semen sample recorded by naked eye and pantone color chips and the corresponding total motile sperm count [file BTM2-6-e10176-s001.pdf]

Table SI. Colorimetric result of the semen sample recorded by naked eye and pantone color chips and the corresponding total motile sperm count

Category: Low TMSC					
Sample No.	No. 1	No. 2	No. 3	No. 4	No. 5
Color	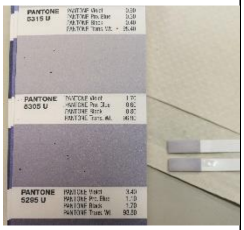	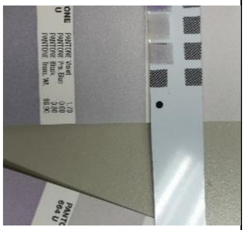	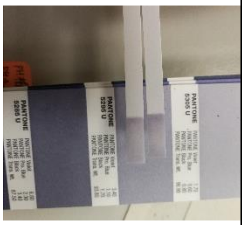	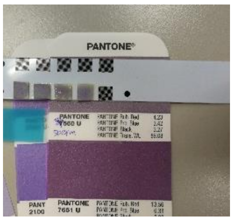	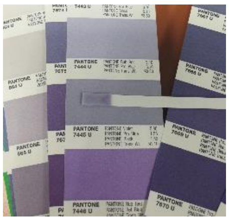
TMSC	0	0	0	0	1
RGB	177, 175, 195	166, 153, 192	177, 175, 195	157, 150, 168	188, 189, 226
Result	✗	✗	✗	✗	✓
Sample No.	No. 6	No. 7	No. 8	No. 9	No. 10
Color	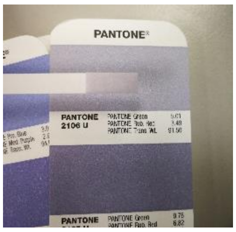	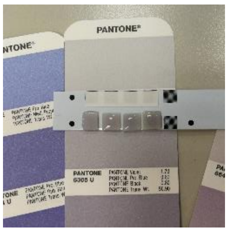	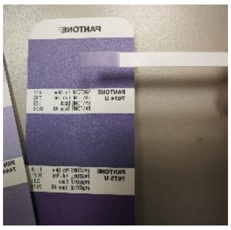	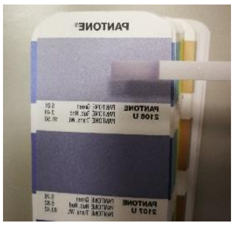	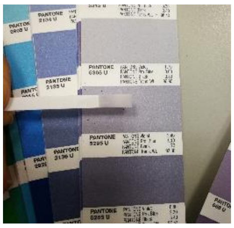
TMSC	0	0	0	0	0
RGB	180, 185, 215	197, 194, 210	146, 150, 184	180, 185, 210	219, 216, 225
Result	✓	✓	✗	✓	✓
Sample No.	No. 11	No. 12	No. 13	No. 14	No. 15
Color	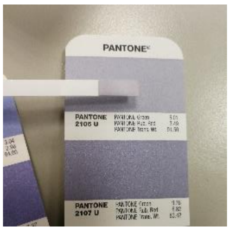	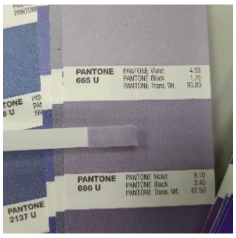	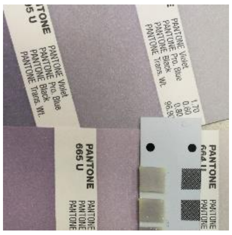	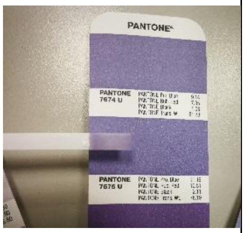	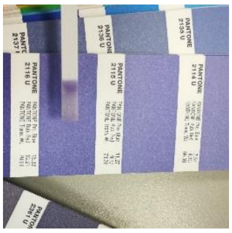
TMSC	0	0	0	0	0
RGB	180, 185, 210	174, 161, 188	197, 184, 206	137, 140, 175	122, 129, 187
Result	✓	✗	✓	✗	✗
Sample No.	No. 16	No. 17	No. 18	No. 19	No. 20
Color	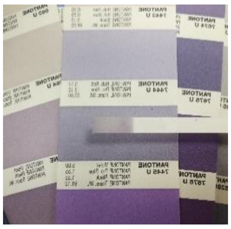	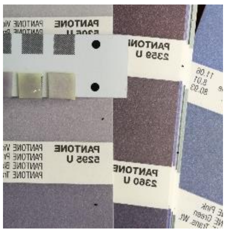	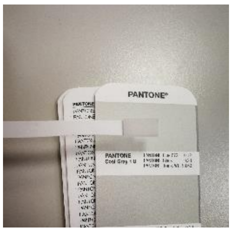	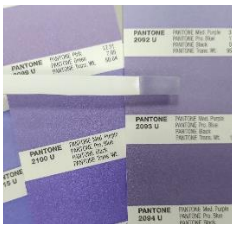	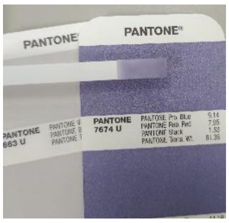
TMSC	0	3	15	13	13
RGB	173, 168, 199	197, 194, 210	218, 217, 215	165, 153, 192	146, 150, 184
Result	✗	✓	✓	✗	✗
Sample No.	No. 21	No. 22			

Color	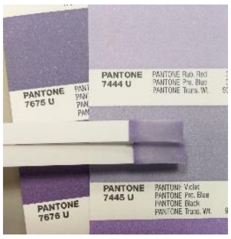	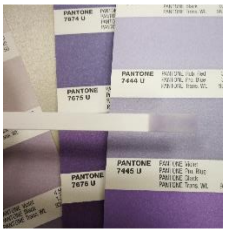			
TMSC	8.7	3.3			
RGB	133, 134, 198	172, 168, 199			
Result	✗	✗			
Category: Normal TMSC					
Sample No.	No. 23	No. 24	No. 25	No. 26	No. 27
Color	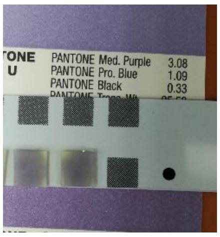	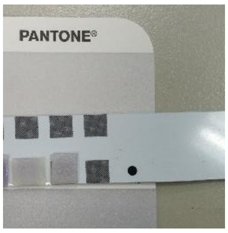	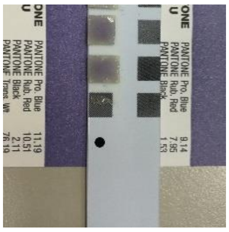	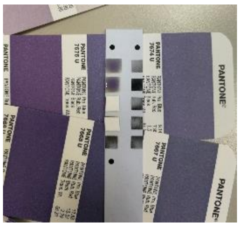	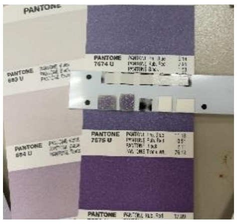
TMSC	67	56	248	86	775
RGB	165, 153, 192	197, 194, 210	137, 140, 175	123, 128, 164	137, 140, 175
Result	✓	✗	✓	✓	✓
Sample No.	No. 28	No. 29	No. 30	No. 31	No. 32
Color	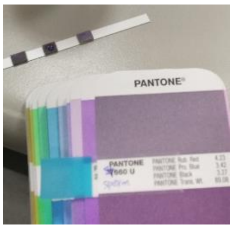	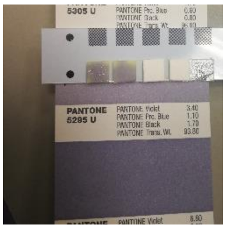	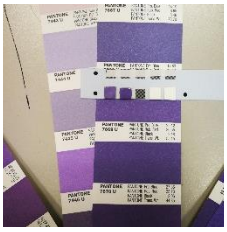	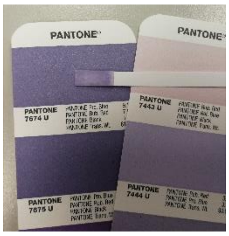	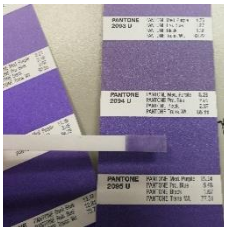
TMSC	67	46	92	122	86
RGB	166, 153, 192	177, 175, 195	138, 136, 181	146, 150, 184	129, 115, 176
Result	✓	✓	✓	✓	✓
Sample No.	No. 33	No. 34	No. 35	No. 36	No. 37
Color	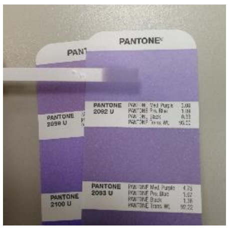	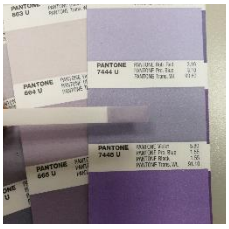	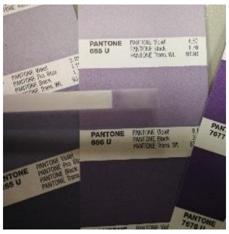	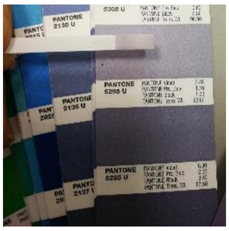	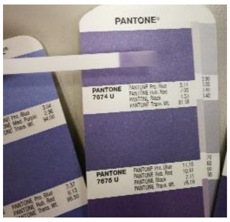
TMSC	266	140	96	74	41
RGB	184, 171, 216	172, 168, 199	174, 161, 188	178, 175, 195	146, 150, 184
Result	✓	✓	✓	✓	✓
Sample No.	No. 38	No. 39	No. 40	No. 41	No. 42
Color	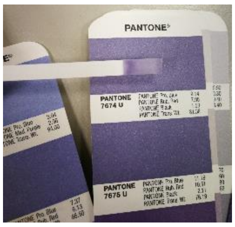	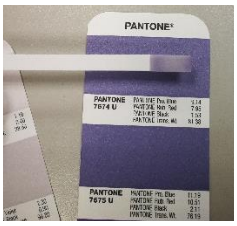	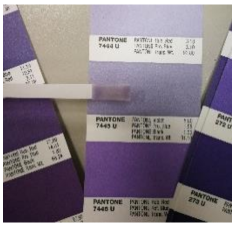	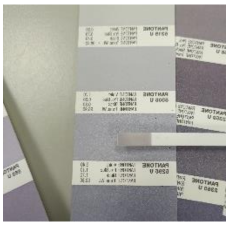	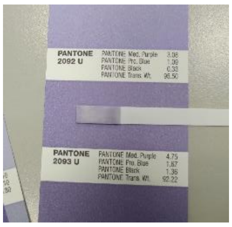

TMSC	36	80	58	27	194
RGB	137, 140, 175	146, 150, 184	146, 150, 184	178, 175, 195	145, 131, 161
Result	✓	✓	✓	✓	✓

TMSC: total motile sperm count; RGB: Red, Green, Blue
