# Supplementary material for: Point‐of‐care semen analysis of patients with infertility via smartphone and colorimetric paper‐based diagnostic device
Source: Bioeng Transl Med. 2020 Aug 18;6(1):e10176. doi: 10.1002/btm2.10176 (PMC7823130; doi:10.1002/btm2.10176)
Supplement: Supplementary file 2 — Table SII Colorimetric result of the semen sample interpreted by the smartphone and the corresponding total motile sperm count [file BTM2-6-e10176-s002.pdf]

Table SI. Colorimetric result of the semen sample interpreted by the smartphone and the corresponding total motile sperm count

Category: Low TMSC

Sample No.	1	2	3	4	5	6	7	8	9	10	11	12	13	14	15	16	17	18	19	20	21	22
Test strip color																						
TMSC	0	0	0	0	1	0	0	0	0	0	0	0	0	0	0	0	3.2	15	14	13	8.7	3.3
Result	✓	✓	✓	✓	✗	✓	✓	✓	✓	✓	✓	✓	✓	✓	✓	✓	✓	✓	✓	✓	✓	✓

Category: Normal TMSC

Sample No.	23	24	25	26	27	28	29	30	31	32	33	34	35	36	37	38	39	40	41	42
Test strip color																				
TMSC	67	56	248	86	775	67	46	92	122	86	266	140	96	74	41	36	80	58	27	194
Result	✓	✗	✓	✓	✓	✓	✓	✓	✗	✓	✗	✗	✗	✗	✓	✓	✓	✓	✗	✓

TMSC: total motile sperm count

*All the sample colors are changed to the gray scale and subtracted by the blank value
